# Singular Effects of a Hæmorrhagic Diathesis

**Published:** 1852-07

**Authors:** N. S. Davis

**Affiliations:** Chicago, Ill.


ART. V.—*Singular effects of a Hæmorrhagic Diathesis.* By N. S. DAVIS, M.D., Chicago, Ill.

IN looking over the paper of Dr. Palmer on "Spontaneous Hæmorrhage," contained, in part, in the June No. of the *Journal*, I was reminded of a case to which I was called some two weeks since.

The patient was a young man, aged about 18 years, and by trade a stone cutter. I found him in bed; his whole aspect—face, skin, lips and tongue—was pale and almost bloodless, with that peculiar sallow hue so common in extreme anæmia. His pulse was 90 per minute, quick and sharp, but easily compressed; tongue clean and moist; appetite impaired; bowels slightly constipated; restless, with occasional slight febrile paroxysms, preceded by chilliness. I could detect no internal visceral enlargement or special derangement of important organs, although the outward aspect was very much like one who had suffered long from extensive enlargement of the spleen, and ague. On examining the posterior part of the right side, I found a very large swelling, extending from under the scapula above to the inferior margin of the ribs, and from the axilla to the spine; lifting the inferior angle of the scapula from one to two inches from the ribs, and extending under the latissimus dorsi muscle. Along the superior and posterior margin of the swelling there was considerable ecchymosis or extravasation of blood, giving the appearance of having been bruised; but there was little pain and no signs of inflammation in the parts. To the touch, the tumor was elastic, tense, and semi-fluctuating, as though it was filled with a jelly or semi-fluid mass.

On enquiry, I learned that he had been subject to excessive hæmorrhages on the slightest cut or injury, and to frequent and profuse epistaxis from childhood. About one year since, a swelling, similar in appearance to the one on his side, presented itself in the calf of the left leg. The swelling gradually disappeared, but leaving the surface over it still ecchymosed and purple. The present swelling made its appearance rather suddenly, and reached its present size in only three or four days, without being preceded by any strain or other injury. From the absence of inflammation, with the semi-fluctuating feel of the swelling, and the history of the case as already stated, I have no doubt but the tumor was produced by the effusion of blood beneath the scapula and the muscles connecting it with the spine, the serum of which was rapidly absorbed, leaving a large clot that still remains.

During my practice I have met with several cases presenting a well marked hæmorrhagic diathesis, but in no instance have I previously found the hæmorrhage to take place spontaneously into the tissues so as to produce true bloody swellings, as in the case just described. As our patient is now under treatment, I shall defer any remarks on its pathology until a future time.

CHICAGO, June 28, 1852.

